# Lightweight Cellulose/Carbon Fiber Composite Foam for Electromagnetic Interference (EMI) Shielding

**DOI:** 10.3390/polym10121319

**Published:** 2018-11-28

**Authors:** Ran Li, Huiping Lin, Piao Lan, Jie Gao, Yan Huang, Yueqin Wen, Wenbin Yang

**Affiliations:** College of Material Science and Engineering, Fujian Agriculture and Forestry University, Fuzhou 350002, China; 18450077642@163.com (H.L.); lanpiao156@163.com (P.L.); gaojiefafu@163.com (J.G.); YH98254@163.com (Y.H.); szylr23@163.com (Y.W.)

**Keywords:** functional, biomaterials, composites, EMI, cellulose foam

## Abstract

Lightweight electromagnetic interference shielding cellulose foam/carbon fiber composites were prepared by blending cellulose foam solution with carbon fibers and then freeze drying. Two kinds of carbon fiber (diameter of 7 μm) with different lengths were used, short carbon fibers (SCF, *L*/*D* = 100) and long carbon fibers (LCF, *L*/*D* = 300). It was observed that SCFs and LCFs built efficient network structures during the foaming process. Furthermore, the foaming process significantly increased the specific electromagnetic interference shielding effectiveness from 10 to 60 dB. In addition, cellulose/carbon fiber composite foams possessed good mechanical properties and low thermal conductivity of 0.021–0.046 W/(m·K).

## 1. Introduction

With the development of science and technology, mankind has developed a variety of electronic technologies, such as broadcasting, television, and microwave technology, which have greatly improved human living conditions. However, these technologies are accompanied by electromagnetic pollution [1,2,3,4], which causes huge damage that cannot be underestimated. If the human body is in an electromagnetic wave environment for a long time, it easily leads to DNA mutations in the body, which may lead to various diseases [5,6].

Based on the shielding principle of electromagnetic radiation, researchers have conducted many studies on electromagnetic shielding materials [7,8,9], and have made many achievements. Ma et al. [10] prepared a novel iron–aluminum sandwich structural composite by hot pressing and subsequent diffusion treatment. The layers were well connected, and its electromagnetic shielding effectiveness could reach 70–80 dB at frequencies from 30 kHz to 1.5 GHz. Joshi et al. [11] achieved electromagnetic shielding effectiveness of up to 60 dB by preparing graphene nanobelt/polyvinyl alcohol film composites. Chen et al. [12] added NaBH_4_ solution to reduce graphite oxide, and deposited the reduced graphite on the surface of carbon fibers by electrophoretic deposition. Its electromagnetic shielding effectiveness reached up to 37.8 dB (8.2–12.4 GHz). Al-Saleh [13] mixed carbon nanotubes, graphite nanosheets, polypropylene (PP), and PE in proportions, and prepared electromagnetic shielding composites by melt blending. The results showed that one-dimensional carbon nanotubes were more effective than two-dimensional graphite nanoplatelets. The electromagnetic shielding performance was good, and the surface adhesion of the polyethylene and carbon nanotubes was also better. Such materials with good electrical properties have potential applications in electromagnetic interference shielding [14]. Carbon nanotubes, graphite, carbon fiber, etc. are commonly used.

Foam materials are also very interesting in EMI shielding research. Ameli et al. studied the shielding effectiveness (SE) property of a foamed PP-CNF (carbon nanofiber) composite. Foaming reduced the density and improved the electrical properties of the composite, which resulted in the increase of the specific EMI SE by up to 65% [15]. Polymer foams reinforced with electron conductive fillers, such as polystyrene-CNT (carbon nanotube) foam [16] composites, graphene reinforced polymethyl methacrylate (PMMA) foam composites [17], lightweight microcellular polyetherimide (PEI)-graphene nanocomposite foams [18], ultralight polyurethane-silver nanowire composites [19], and so on, have been shown to exhibit high EMI SE at very low densities. However, the matrices of EMI foam materials are always resin and plastic, which are not environmentally friendly.

In previous work, ultra-light-weight cellulose foams were realized by combination of the foam forming technique and a novel cellulose solvent by adding sodium dodecyl sulfate (SDS) to the NaOH/urea aqueous solution with a further mechanical stir [20]. Cellulose foam is ultra-low density and has good mechanical properties; as a result, it can be utilized as a matrix to fabricate composite materials via mixing with some other functional fillers. Here, we want to develop an easy way to manufacture electromagnetic shielding foam, which blends cellulose foam and carbon fiber together.

## 2. Experimental Section

### 2.1. Materials

The cellulose was provided by Hubei Chemical Fiber Group Ltd. (Xiangfan, China) in the form of cotton linter pulp, in which the α-cellulose content was more than 95%. The cellulose needed to be pretreated before using—washed in distilled water and then oven dried for 24 h. The viscosity-average molecular weight (*M_η_*) of cellulose in cadoxen was determined, using an Ubbelohde viscometer at 25 °C, to be 9.6 × 10^4^ (degree of polymerization, *DP* = 600), according to the Mark-Houwink equation [*η*] (mL·g^−1^) = 3.85 × 10^−2^ (*M_w_*)^0.76^ [21]. Carbon fibers were provided by Kingfa Science & Technology Co. Ltd. (Guangzhou, China). All other chemical reagents, such as sodium dodecyl sulfate (SDS) and sulfuric acid, were purchased from Shanghai Chemical Reagent Co., Ltd. Shanghai, China, and were of analytical grade.

### 2.2. Preparation of Composite Cellulose Foams

**Figure 1:**
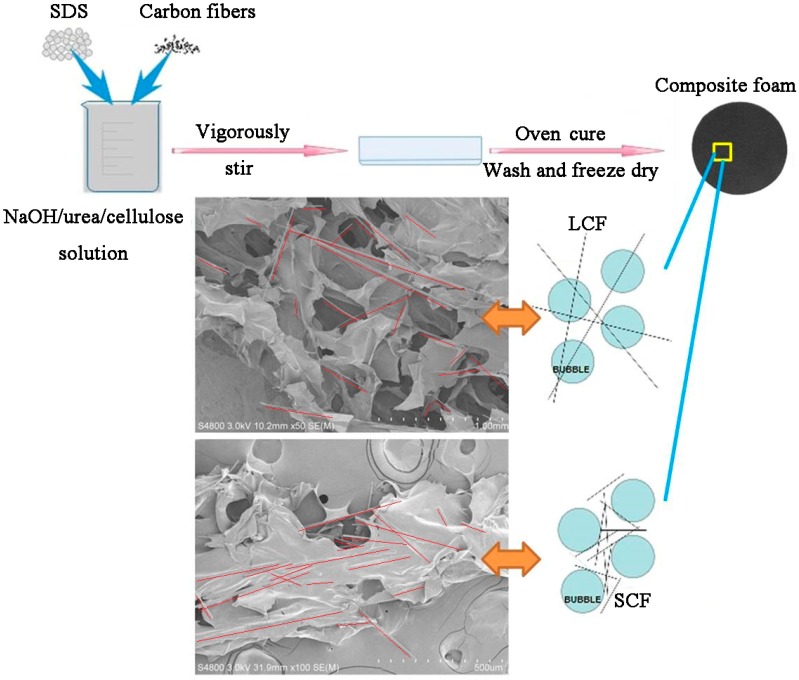
Schematic diagram of the production process of the composite foams, and of the foam structure.

Cellulose solution was prepared according to a previous method [22]. Fourteen grams of NaOH, 24 g urea and 162 g distilled water were added to a 250 mL beaker to produce a mixed aqueous NaOH/urea solution. The solution was then frozen until the temperature reached −12.5 °C. After that, 8.4 g pretreated cotton linter pulp was immediately added into the precooled solution and stirred vigorously for 5 min at room temperature, resulting in 4 wt % transparent cellulose solution. The cellulose solution was centrifuged at 7200 rpm for 15 min at 10 °C to remove the small remaining undissolved part, impurities, and bubbles. As shown in Figure 1, 2 g sodium dodecyl sulfate (SDS) and a certain amount of carbon fibers (5%, 10%, 15%, 20% of cellulose) were mixed into the transparent NaOH/urea/cellulose aqueous solution. After 30 min vigorous agitation at room temperature, a bubble solution was produced and then poured into a cylindrical tube. The cellulose foams were formed via heating at 60 °C for 4 h. The resulting foams were washed with running water and then distilled water until neutral, and finally freeze dried. Foams were coded as SCFxx and LCFxx, where xx was the carbon fiber content.

### 2.3. Characterization

Scanning electron micrographs (SEM) were taken on a Hitachi S4800 scanning electron microscope (Hitachi, Tokyo, Japan) with 3 kV accelerating voltage, at magnification of 50 and 5000 respectively. The foams in their wet state were treated in liquid nitrogen, immediately snapped and then freeze dried. The samples were sputtered with gold, then were observed and photographed. Pore size was read from the SEM images. FT-IR spectra were recorded on a Nicolet FTIR spectrometer (Nicolet NEXUS 670, Thermo, America) by KBr pellet method. The dried cellulose foams were cut into cubes to measure the volume (*V*) and the mass (*m*), and then the density (*ρ*) was calculated through the equation: *ρ* = *m*/*V*.

Testing for the EMI shielding effectiveness of the present composite cellulose foam samples was conducted at 25 °C over an emission frequency range of 30–1500 MHz, using the DR-S01 shielding effectiveness tester, produced by Beijing Dingrong Shichuang Science & Technology Co. Ltd. (Beijing, China). The electrical conductivity of composites was measured using the four point method on a resistivity/Hall measurement system (Scientific Equipment & Services, USA). The contact points of the samples were coated with silver paste to reduce the contact resistance. Voltage and current data was recorded after the display became stable following full electrical connections between the probes and composites. At least five measurements were made for each set of conditions.

The tensile strength (*σ_b_*) and compression properties of the foams were measured on a universal testing machine (CMT6503, Shenzhen SANS Test Machine Co. Ltd., Shenzhen, China) according to ISO 527-2, 1993 (E) at a speed of 5 mm·min^−1^, respectively. The *σ_b_* values recorded were the average of five measurements.

## 3. Results and Discussion

### 3.1. Fabrication of Cellulose Composite Foams

**Figure 2:**
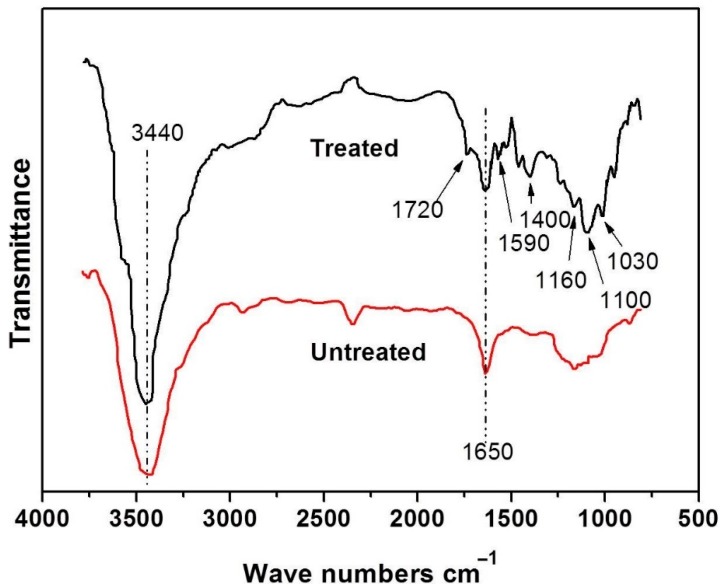
FT-IR spectrum of raw carbon fiber and treated carbon fiber.

In this research, two kinds of carbon fiber, *L*/*D* rates of 100 (SCF) and 300 (LCF) respectively, were added into cellulose foam to create electromagnetic shielding and insulation foam. Carbon fibers were treated with 98 wt % H_2_SO_4_ for several hours to remove hydrophobic reagents on the surface, and oxidize the carbon fiber to make it more hydrophilic. These changes were measured by FTIR, which is shown in Figure 2. The lower curve is the absorption spectrum of the untreated carbon fiber, and the upper curve is the carbon fiber absorption spectrum after the treatment. It can be seen that after electrochemical treatment, the carboxyl characteristic peak ν_COOH_ appears near 1720 cm^−1^, and there is a characteristic peak of the carboxylate (COO–) group near 1590 cm^−1^, indicating that the surface of the carbon fibers was attached after the sulfuric acid treatment. The peak intensity at 1650 cm^−1^ is derived from the hydrogen bond in the acid [23]. A weak absorption peak appears near 1400 cm^−1^, which possibly due to carboxyl coupling vibration and hydroxyl deformation vibration. The characteristic peak absorption of the hydroxyl group near 3440 cm^−1^ is also greatly enhanced, indicating that the number of hydroxyl groups is greatly increased. A main peak splitting into several small peaks near 1160 to 1030 cm^−1^ may be the stretching vibration absorption peak of C–O in different groups such as carboxyl group, lactone group, and phenol group, and the small absorption peak at 1160 cm^−1^ is the stretching vibration absorption peak of the C–O bond in the carboxyl group. The small absorption peak at 1060 cm^−1^ is the absorption peak of the C–O group in the lactone group, and the peak appearing at 1030 cm^−1^ may be the stretching vibration absorption peak of the CO group in the phenol group. It is indicated that after electrochemical oxidation treatment, the reactive functional groups attached to the surface of the carbon fibers are carboxyl groups, hydroxyl groups, and lactone groups, which will be compatible with cellulose.

**Figure 3:**
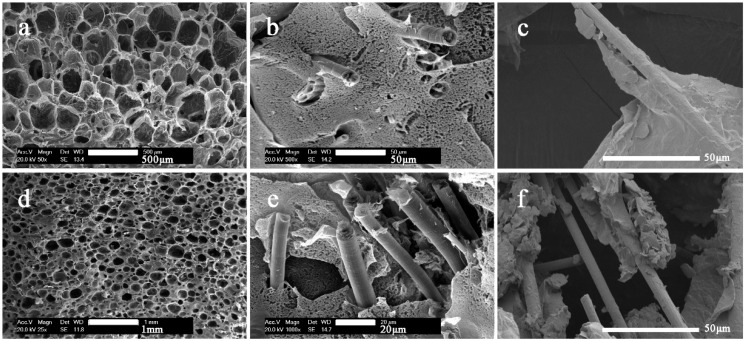
SEM images of the composite foams. (**a**–**c**) SCF15 at different magnification; (**d**–**f**) LCF15 at different magnification.

After acid treatment, the carbon fibers were added to cellulose solution to prepare composite foams. Figure 3 shows the SEM images of the foams with long carbon fibers (LCF) and short carbon fibers (SCF) at different magnification. Both fibers were well coated by cellulose, which revealed a good compatibility between carbon fiber and cellulose. The introduction of carbon fiber was found to increase the bubble cell size of the composite foams, as shown in Table 1. In pure cellulose foam, cell size is about 105 μm, which is much shorter than carbon fibers. Thus, with addition of carbon fiber, bubbles were more likely to be pushed together by carbon fibers, which caused the bubbles to merge and collapse during the process of bubble solution forming cellulose foam. We took some specimens inside the foam, and the SEM images are shown in Figure 1. Carbon fiber network structure was gradually formed. Most of the SCFs were in cellulose lamellae. That might be because the cell size was about several hundred micrometers, which is almost the same the length as the SCFs. Moreover, some exposed LCFs were found. It is obvious that bubbles were strung on LCFs, as the carbon fibers were long enough, which made the fiber network structure more efficient. The disadvantage is that the bubbles are more likely to merge and collapse with the increased content of carbon fiber, which will affect the density and thermal conductivity of the foams. The density of the foams was changed greatly when the carbon fiber content was increased, as shown in Table 1.

### 3.2. EMI Shielding of Cellulose/Carbon Fiber Composite Foams

**Figure 4:**
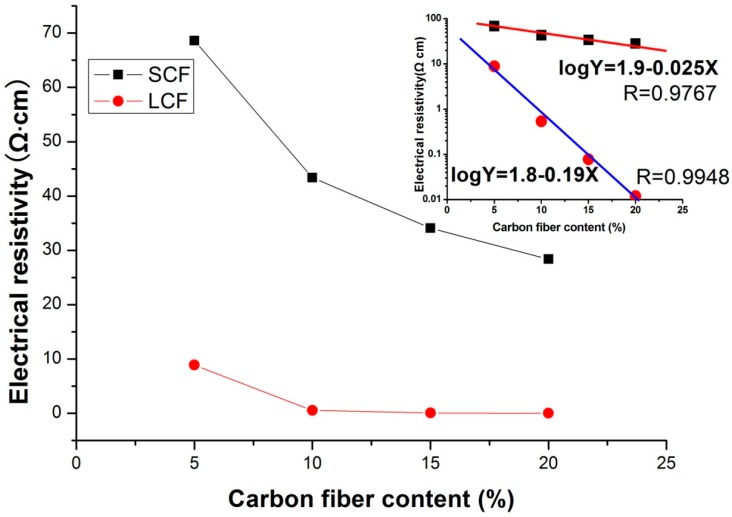
Electrical resistivity for the composite foams as the function of carbon fiber content.

Electrical conductivity is critical for EMI shielding efficiency, because it is an intrinsic ability of a material for absorbing electromagnetic radiation [24]. As shown in Figure 4, with the increase of carbon fiber, the electrical conductivity of the foams was better. LCF20 showed the best conductivity, which was 0.012 Ω∙cm.

In general, the foaming process has two effects on the electrical conductivity of polymer composites [18]. One effect is that the excluded volume, related to bubble formation, pushes fillers (SCF and LCF) together; even more important, the strong extensional flow generated in situ during bubble growth facilitates the orientation of fiber fillers in bubble cell wall [25]. The enriching and orientation of fibers causes the fibers in the foamed composites to pack closely. The other effect of the foaming process is volume expansion, which tends to increase the distance of adjacent fibers [26]. As we see in the SEM images in Figure 1, short carbon fibers are most likely oriented in cellulose lamellae between the bubbles, and long carbon fibers can easily penetrate through the bubbles (carbon fibers are marked by red line). So, LCFs more easily build conductive networks in the composite foam, which gives LCF foams good electrical conductivity, as shown in the schematic structure insert in Figure 1.

**Figure 5:**
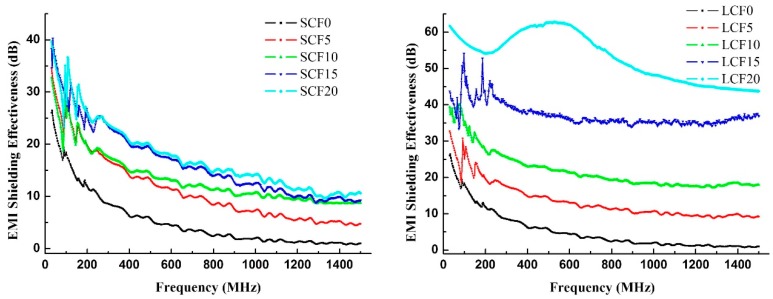
EMI shielding effectiveness of the composite foams at the frequency range of 30–1500 MHz.

The malfunction of electronics can be hazardous, as the electronics can be associated with strategic systems such as aircraft, nuclear reactors, transformers, control systems, communication systems, etc. [27]. Figure 5 presents the EMI SE of the foams over a frequency range of 30 to 1500 MHz. Pure cellulose foam is less than 20 dB, almost 0 dB at high frequencies, which indicates that pure cellulose foam has no EMI SE because pure cellulose is an electrical insulator. With the increase of SCF, the EMI SE of the foams increased to 10 dB (high frequency). When SCF content was changed from 10% to 20%, the EMI SE slightly increased. However, as the LCF increases, the EMI SE of the foam increases from 0 dB to over 45 dB at high frequencies, especially in the range of 400 to 700 MHz, where the LCF20 reaches 60 dB. The reason for the difference in EMI SE between the two samples was mainly due to the obvious decrease in electrical resistivity of LCF foams, as we have discussed. EMI depended on the electrical conductivity of the foams. LCF foams, especially LCF20, exhibited better electrical conductivity and thus their EMI was higher.

**Figure 6:**
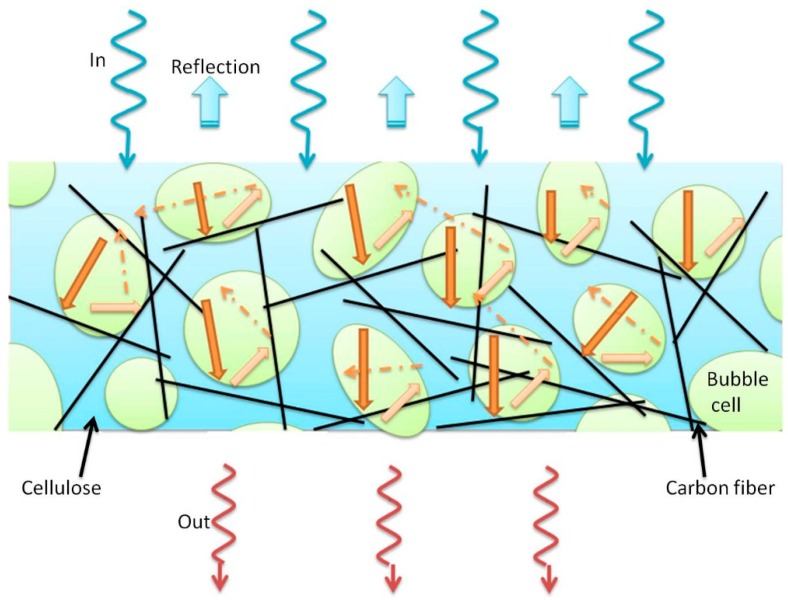
Schematic description of the microwave transfer across cellulose/carbon fiber composite foam.

According to the results, SCFs were oriented in cellulose lamellae and separated by bubbles, which made the network not so good. LCFs connected with each other directly to make the conduct network good even at low content. Good network means larger electron conductivity, leading to better EMI properties. Furthermore, it was proposed that the spherical air bubbles in the foam structure enhanced the attenuation of incident electromagnetic microwaves by multiple reflection and decay between the cell wall and fillers [28]. As indicated in Figure 6, the spherical microscale air bubbles in the foams could attenuate the incident electromagnetic microwaves by reflecting and scattering between the bubble lamellae and fillers, and it was difficult for the microwaves to escape from the sample before being absorbed and transferred to heat [18].

### 3.3. Thermal and Mechanical Properties of Cellulose/Carbon Fiber Composite Foams

**Figure 7:**
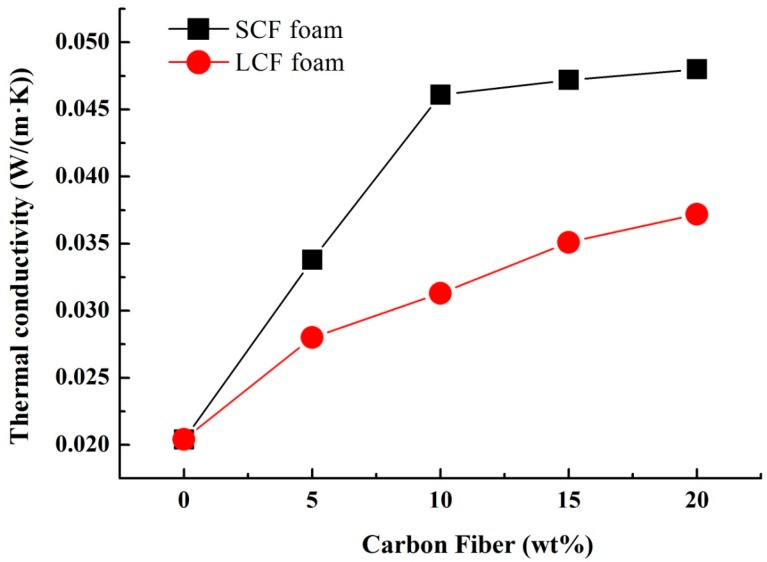
Thermal conductivity of the composite foams.

Foams always have good thermal insulation properties, which is quite useful in the area of building materials. The thermal conductivity of the foams is shown in Figure 4. Low thermal conductivity reveals good thermal insulation. The pure cellulose foam showed the lowest thermal conductivity 0.021 W/(m·K), which revealed the best insulation. With the increase of carbon fiber content, the thermal conductivity of the foams increased. There are two reasons: one is the burst of the bubbles increases the density of the foam, as we seen in Figure 7; the other one is that the thermal conductivity of carbon fiber is greater than that of cellulose. Interestingly, thermal insulation of LCF foams was better than SCF foams. The main reason is that long carbon fibers tend to form a network structure and prevent shrinkage when bubbles are broken, leading to a low density. As a result, the cellulose foams are good thermal insulation material.

**Figure 8:**
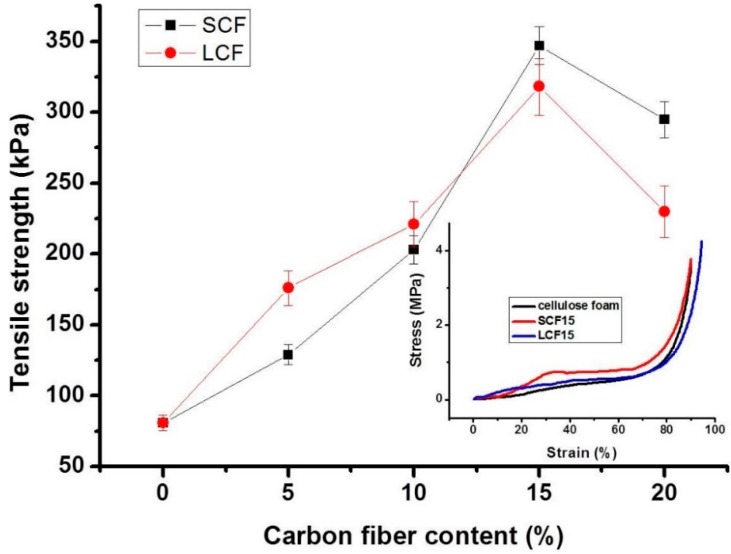
Mechanical properties of the cellulose composite foams as a function of carbon fiber content. Insert figure—compressive strength of the foams as a function of strain.

Figure 8 shows the mechanical properties of the composite foams. The tensile strength of pure cellulose foam was about 80 kPa. With the increase of carbon fiber loading, the tensile strength of cellulose / carbon fiber composite foams increased to 350 kPa (SCF) and 320 kPa (LCF) both at 15 wt % as a result of carbon fiber enhancement. A further increase in carbon fiber content decreases the tensile strength to 300 kPa (SCF) and 240 kPa (LCF). Large pores makes LCF much easier to slit. Moreover, parts of LCFs exposed inside bubble cells make the tensile strength enhancement less than that of the oriented SCFs at high carbon fiber content. Compression property of cellulose foams are shown insert Figure 8. All the cellulose foams displayed typical “J” shape curves. Though the compression strain was over 90%, the compression stress still went up. The results also indicated that the porosity of the foams was over 90%.

## 4. Conclusions

In summary, we have developed an easy and fast approach for scalable fabrication of lightweight cellulose/carbon fiber composite foam, based on a cellulose dissolution and regeneration process. Acid treated short carbon fibers (SCF) and long carbon fibers (LCF) were added into the cellulose solution to produce EMI composite foams. Carbon fibers built conductive networks, where SCFs were most likely oriented in the bubble cell wall and LCFs penetrated through the bubbles. LCF/cellulose foams showed better electrical conductivity and higher EMI shielding property (60 dB of LCF20). Furthermore, cellulose/carbon fiber composite foams exhibited well-defined thermal insulation and tensile properties. The comprehensive study of cellulose composite foams based on other powerful absorbers will be required in the future, in order to develop useful materials for EMI shielding in high-tech fields.

## Figures and Tables

**Table 1:** Density and cell size of the composite foams.

Foams	Density (mg/cm^3^)	Cell Size (μm)	Foams	Density (mg/cm^3^)	Cell Size (μm)
CF	33	105 ± 25			
SCF5	36.2	117 ± 33	LCF5	35.5	129 ± 34
SCF10	48.5	141 ± 35	LCF10	39.9	165 ± 40
SCF15	62.1	181 ± 39	LCF15	46.3	217 ± 52
SCF20	79.3	238 ± 46	LCF20	57.8	301 ± 68
